# Ecological momentary intervention to enhance emotion regulation in healthcare workers via smartphone: a randomized controlled trial protocol

**DOI:** 10.1186/s12888-022-03800-x

**Published:** 2022-03-05

**Authors:** Diana Castilla, María Vicenta Navarro-Haro, Carlos Suso-Ribera, Amanda Díaz-García, Irene Zaragoza, Azucena García-Palacios

**Affiliations:** 1 Department of Personality, Evaluation and Psychological Treatment, University of Valencia, Avenida Blasco Ibáñez, 21, 46010 Valencia, Spain; 2 CIBER of Physiopathology of Obesity and Nutrition (CIBEROBN), ISCIII CB06/03/0052, Instituto Salud Carlos III, 28029 Madrid, Spain; 3 Department of Psychology and Sociology, University of Zaragoza, Calle Atarazana, 4, 44003 C/ Ciudad Escolar, s/n, 44001 Teruel, Spain; 4 Instituto de Investigación Sanitaria Aragón, Avenida de San Juan Bosco, 13, 50009 Zaragoza, Spain; 5 Department of Basic Psychology, Clinical Psychology and Psychobiology, Universitat Jaume I, Avenida de Vicent Sos Baynat, s/n, 12071 Castellón de la Plana, Spain

**Keywords:** Emotion regulation, Transdiagnostic, Ecological momentary intervention, Smartphone, RCT, Health professional, Intervention, APP, CBT, DBT

## Abstract

**Background:**

CUIDA-TE is an APP that offers transdiagnostic cognitive behavioral therapy focused on enhancing emotion regulation. As a novelty, it incorporates ecological momentary interventions (EMI), which can provide psychological support in real time, when suffering arises. The main goal of the study is to evaluate the efficacy of CUIDA-TE to improve emotion regulation in healthcare workers, a population that has been particularly emotionally impacted by the COVID-19 pandemic.

**Methods:**

In this three-arm, randomized controlled trial (RCT) the study sample will be composed of a minimum of 174 healthcare workers. They will be randomly assigned to a 2-month EMI group (CUIDA-TE APP, *n* ≥ 58), a 2-month ecological momentary assessment (EMA) only group (MONITOR EMOCIONAL APP, n ≥ 58), or a wait-list control group (no daily monitoring nor intervention, n ≥ 58). CUIDA-TE will provide EMI if EMA reveals emotional problems, poor sleep quality/quantity, burnout, stress, or low perceived self-efficacy when regulating emotions. Depression will be the primary outcome. Secondary outcomes will include emotion regulation, quality of life, and resilience. Treatment acceptance and usability will also be measured. Primary and secondary outcomes will be obtained at pre- and post-intervention measurements, and at the 3-month follow-up for all groups.

**Discussion:**

To our knowledge, this is the first RCT that evaluates the efficacy of an APP-based EMI to improve emotion regulation skills in healthcare workers. This type of intervention might ultimately help disseminate treatments and reach a larger number of individuals than traditional face-to-face individual therapies.

**Trial registration:**

ClinicalTrial.gov: NCT04958941 Registered 7 Jun 2021.

**Study status:**

Participant recruitment has not started.

**Supplementary Information:**

The online version contains supplementary material available at 10.1186/s12888-022-03800-x.

## Background

### The pandemic and healthcare workers

On March 11th, 2020, the WHO declared a worldwide pandemic level for a new coronavirus, the SARS-COV-2, better known by its disease name: COVID-19 [1]. This pandemic has pushed the health systems around the world, including the Spanish one, to the limit. For example, the Spanish National Institute of Statistics estimates that over 84 thousand individuals have died due to COVID-19 in Spain [2], while over 4.5 million deaths have been calculated globally.

In addition to the burden of COVID-19 on our health systems, experts and institutions around the world have increasingly claimed for the importance of this pandemic on the mental well-being of individuals [3]. The psychological side effects of the COVID-19 pandemic are vast and range from social isolation, fear of infection, marital conflicts, anxiety, post-traumatic stress, and depression, among others [4]. In particular, a group of individuals that have experienced additional burden due to their high exposure to the disease and high work-related demands are healthcare professionals. A recent literature review including 24 studies supports the idea that the psychological effects of the pandemic on healthcare workers has been devastating globally [5]. This is also the case of Spain, where at least 50% of Spanish healthcare professionals are at high risk of developing a mental disorder as a result of the stress associated with COVID-19 [6]. In particular, this study showed that 28.1% of healthcare professionals suffered from depression, 22.5% had an anxiety disorder, almost 1 in 4 suffered from panic, 22.2% had post-traumatic stress disorder, and over 6% suffered from substance abuse. In addition, a recent investigation revealed that up to 8.4% of healthcare professionals have experienced suicidal ideation during the pandemic [6].

Healthcare workers are and have been facing very severe work stressors for many months. For example, they have been fighting against a relatively unfamiliar virus, have had to work for longer hours than usual, have often faced a high work overload and significant risk of infection, have had to follow very strict safety protocols, and have been asked to remain highly concentrated and vigilant [7, 8]. Not surprisingly, burnout and fatigue have significantly increased in this population as a result of the COVID-19 crisis [9]. In this line, a study conducted at the beginning of the pandemic revealed that the most common symptoms in Spanish healthcare professionals were stress, anxiety, depression, and insomnia, which all occurred in significant levels [10]. In another large study conducted during the first wave of the COVID-19 pandemic, one in seven healthcare professionals in Spain screened positive for a disabling mental disorder, from which emotional disorders were the most frequent [11]. The findings suggested that workers frequently exposed to patients affected by COVID-19 patients or those who have been infected or quarantined/isolated should be considered groups that need special monitoring of mental health and psychological support. In addition to these problems, and similar to past epidemics, other adverse psychological reactions have also been observed, such as fear of infection, fear of contagion, fear of infecting family and friends, and uncertainty and stigmatization [12–14], which again adds to the burden of the COVID-19 pandemic.

As a result of the previous, there is growing concern about the psychological adjustment and recovery of healthcare workers who directly treat and care for patients, especially when the latter have an active infection or belong to vulnerable populations (e.g., the elderly). In particular, researchers support the idea that, if not adequately and timely addressed, the aforementioned stressors associated with patient care during the pandemic could potentially lead to the development of long-term anxiety disorders, depression, or post-traumatic stress disorder [10]. As a result, recent studies have concluded that it is necessary to offer healthcare professionals, especially those who have been more impacted by the pandemic, psychological help to reduce the emotional impact of COVID-19. Ultimately, this might not only benefit their mental health, but also it will protect and help maintain the sustainability of our healthcare systems [15–18].

### Transdiagnostic interventions to address emotion regulation in persons with emotional problems

In the last decade, transdiagnostic treatments have gained ground in the treatment of psychological problems, particularly anxiety and depression problems (i.e., emotional disorders, ED). Transdiagnostic treatments emphasize the need to target essential, shared mechanisms or processes that are common to a wide range of emotional problems without tailoring the protocol to specific diagnoses [19]. This is important because it allows to offer a single intervention to individuals with different emotional problems, even if they have comorbidities (i.e., more than one emotional problem).

A transdiagnostic intervention for EDs that has received particular attention in the past years is the Unified Protocol [20]. According to the UP, individuals with emotional disorders share a tendency to frequently and intensely experience negative affect and exhibit maladaptive reactions to these intense, unpleasant emotions [21, 22]. These responses to difficult emotional states are known as emotion regulation (ER) efforts and are key underlying mechanisms of psychopathology for the majority of third-wave psychological interventions, such as Dialectical Behavior Therapy (DBT [23, 24];), Acceptance Commitment Therapy (ACT [25];), and the Unified Protocol [20]. In fact, a recent systematic review [26] identified ER components across all intervention modalities and disorders, thus indicating that ER may be a transdiagnostic factor involved both in the expression and treatment of psychopathological problems. Vast evidence now supports this idea of ER as a transdiagnostic construct [27–29].

ER is a complex process that involves regulating emotional arousal and emotional expressions flexibly according to the context demands [30, 31]. Research has shown that emotion regulation strategies are associated with burnout in the professionals, so emotion regulation training is fundamental for healthcare professionals [32]. When people deal with stress, such as the one described in healthcare professionals during the COVID-19 pandemic, ER enables individuals to evaluate the emotional impact of the situation and helps them to make decisions about the most preferable emotional reactions in that situation [33]. In particular, the UP focuses on regulating negative affect by addressing emotional avoidance, promoting cognitive flexibility, facilitating exposure to avoided situations and sensations, and increasing present-focused emotional awareness [34].

Another treatment approach that includes principles or components that could be useful to target difficulties in emotion regulation is DBT. DBT is based on a skill deficit model that views dysfunctional behavior as either a consequence of dysregulated emotions or a maladaptive approach to ER [24]. DBT places a special emphasis on increasing experiential awareness and acceptance (mindfulness techniques), but it also includes behavioral strategies focused on changing emotional reactions (e.g., opposite action) [35]. This treatment has been suggested to be effective for a wide range of disorders with symptoms that are functionally similar to those of borderline personality disorder (e.g., depression, substance use disorder, anxiety disorders, and eating disorders), for which emotion dysregulation has been proposed to be the main etiological and transdiagnostic factor [35]. More recently, DBT has also been used to address emotion dysregulation in clinical and non-clinical populations [36].

Considering the aforementioned evidence of transdiagnostic interventions for ER, the treatment proposed in this study will include different strategies from the UP and DBT. An emphasis will be placed on the prevention of emotional problems that may occur due to recurrent stress in healthcare professionals. Based on the results of past research, the selection of prevention strategies targeting common transdiagnostic processes (e.g., emotional dysregulation), as opposed to separate diagnostic categories, would present a number of advantages: [1] increase treatment efficacy, because common prevention strategies targeting shared processes would replace numerous interventions targeting specific disorders, thus allowing to treat more people simultaneously with less resources [2]; reduce economic costs by using only one intervention to prevent a broad set of emotional problems [3]; and, finally, transdiagnostic prevention strategies emphasize improving individual quality of life and functioning, which might help prevent disorders before they occur [34].

### Internet-delivered interventions

While the importance of psychological help during the pandemic has been recognized, healthcare professionals appear to face important barriers and restrictions to receiving mental health support [37]. These appear to include, for example, the stigma associated with mental care, time restrictions, and lack of knowledge about the available resources for help. A recent call to action from mental health science underlines the importance of research identifying interventions that can be delivered under pandemic conditions to reduce its psychological impact, especially on vulnerable populations [38]. Self-applied, online, and brief interventions could be a workable solution in this scenario.

COVID-19 has resulted in an unprecedented acceleration and penetration of the digital world in everyday life in general and in the mental health field in particular [4], so a new era of digital interventions is expected [39]. While acknowledging this boost of information and communication technologies (ICTs) for mental care due to the COVID-19 pandemic, the use of ICTs is not new in the Psychology field. Internet-delivered interventions have demonstrated their efficacy in more than 100 controlled clinical trials [40, 41]. In the last 10 years, due to the increased availability of the Internet worldwide and its ubiquity through smartphones, a remarkable progress has been made in the field of psychological treatments with ICTs. As a result, the inclusion of technologies in psychological care has helped overcome some barriers and obstacles associated with psychological treatments, such as stigma, anonymity, economic accessibility, waiting lists, and travel restrictions, among others [42]. Self-applied, online treatments can reach the patient in a more economical and immediate way and are accessible to people who would otherwise experience difficulties when attempting to receive treatment (e.g., due to long geographic distances to health centers, limited financial resources, or lack of time). Therefore, such interventions are feasible alternatives from an economic point of view, since they allow the dissemination of evidence-based treatments at low costs [43]. Due to the advantages of self-applied, online psychological interventions, the aim of the present study is to test their efficacy and feasibility in a sample of healthcare professionals that have been in the front line of patient care during the COVID-19 pandemic.

### Ecological momentary assessments and interventions: the importance of real time, ecological approaches to ER

Digital treatment platforms have advanced notably in the past years. Mobile devices, such as smartphones, are effective ways to deliver psychological interventions [44] and improve psychological assessment by minimizing problems like recall bias due retrospective assessments and the poor adherence to paper diaries [45]. In addition, mobile devices take the aforementioned advantages of online treatments one step further by opening the door of ubiquity to ICT-based psychological interventions. In particular, smartphones allow users to carry the treatments with them in their pockets, which permits clinicians to react in real or in short time when a problem is detected (e.g., if a patient does not leave the home for several days) and facilitates tailoring (i.e., personalizing) treatments to the patient needs at any given moment (e.g., a treatment component can be provided depending on the problem reported in real time) [42].

Another advantage of mobile devices is that they facilitate the assessment and treatment of ER as situational and context-related processes [46]. ER strategies are not adaptive or maladaptive per se, but dependent on the context in which they occur [47, 48]. For example, research has shown that, when the emotional intensity of a situation is low, people tend to use reappraisal [49], which is a form of cognitive change that involves reinterpreting the meaning of a potentially emotion-eliciting situation [50]. Conversely, in the context of a situation with high emotionality, distraction (that consists of disengaging attention from emotional processing by producing neutral thoughts that are independent from and not in conflict with emotional information [51]) is usually preferred [46]. A possible explanation suggested by Fruzzetti and Shenk [52] is that, when people are highly emotionally aroused, they experience poorer cognitive capacity, less self-awareness, and impaired ability to solve problems, which means that distraction may be an adaptive strategy on that specific context. There is already some evidence in this regard. For example, Sheppes et al. [53] showed that, when the intensity of emotional stimuli was low, cognitive demands were accordingly low, long-term goals were more easily activated, and individuals were more likely to implement cognitive reappraisal as an ER strategy. However, when the emotional stimuli were very intense, cognitive demands were high, short-term goals were more easily activated, and participants were keener to use distraction as a coping strategy. Overall, these findings support the view that ER strategy choice and utility is situational and context-related [46].

A common barrier to evaluating the effectiveness of psychological treatments has been related with the dynamic nature of emotional phenomena, which in a healthy individual fluctuates not only over time, but also notably within a single day [54]. To date, research regarding ER has mostly been based on laboratory experiments, which tend to rely on retrospective evaluations and leave situated and momentary aspects relatively unknown [47]. To overcome these limitations, ecological momentary assessment (EMA) has become increasingly popular in the ER research [55]. EMA, also called Experience Sampling Method (ESM), emerged in the 1980s [56, 57], but has been traditionally difficult to implement due to the limitations of paper diaries [58]. EMA requires the evaluation of individuals in the natural context and the moment (or as close as possible) in which events (e.g., an emotion) occur to increase the reliability and ecological validity of the measure obtained. EMA makes it possible to collect repeated inputs of thoughts, feelings, and behaviors close in time to the experience and in real-life contexts [47].

The popularity of EMA studies has clearly increased in the past years, arguably due to the increased availability of mobile applications [55]. EMA has been used for both subjective [58] and objective [59] data collection, and this method has already significantly increased our knowledge about ER outside laboratory settings [60]. Smartphone devices, which are now more accessible than ever [61], have shed new light into EMA and have renewed interest for this type of methodology.

In addition to EMA, smartphones facilitate Ecological Momentary Interventions (EMI), because they allow to provide psychological care to individuals in their natural environment at the time when the support is needed [62]. EMIs consist of momentary treatments provided via mobile technologies while people are engaged in their typical everyday life routines. These interventions can be used as an adjunct to existing psychological therapies delivered by a therapist or they can be implemented as a stand-alone intervention [63]. EMIs are important because they provide the patients with timely recommendations or therapeutic instructions when problems occur as opposed to time later during onsite appointments. This has been argued to reduce patient suffering, enhance treatment effectiveness, and reduce treatment costs [64, 65], which makes this a very suitable methodology to be implemented in new generation psychological treatments for EDs.

In sum, the availability and spread of smartphones has made EMA and EMI more feasible than ever. In fact, a recent systematic review into psychological interventions using mobile devices revealed an exponential growth of published studies focusing on the development of psychological treatments via smartphones [44]. This study, however, indicated that a large number of investigations have used smartphones for monitoring purposes only (EMA), while RCTs comparing different functionalities or conditions are still infrequent (15.8%). The present investigation represents a step in this direction.

### The current study treatment

As mentioned earlier, healthcare workers have been under a great deal of stress for almost 2 years due the COVID-19 pandemic. As a result, an important number of them have experienced significant levels of stress, anxiety, depression, and insomnia [10]. It is important to note, however, that healthcare workers are not necessarily clinical population with previous psychopathological problems. On the contrary, they might be mentally healthy individuals that are exposed to important chronic stressors and, as a consequence of this exceptional situation, might benefit from some psychological support for prevention purposes. As noted earlier, learning ER skills can prevent the onset of emotional disorders, their maintenance, and worsening, as well as improve the quality of life of individuals. In particular, transdiagnostic interventions focused on teaching emotion regulation skills may be good therapeutic options to teach healthcare professionals how to cope with stress [26] and burnout [32].

Because time restrictions in healthcare professionals are frequent and we aim to offer timely interventions, we propose a two-month brief intervention using smartphones. This technology allows users to receive a treatment in real/short time and facilitates the adaptation of treatments to the needs of patients at any given moment. In this study, we aim to take a step further by comparing two types of innovative interventions. More specifically, the current study will analyze the efficacy of an ecological momentary intervention (EMI) to enhance emotion regulation skills compared with an ecological momentary assessment (EMA), which might enhance awareness of emotions, and a wait-list control group (no daily monitoring nor intervention).

The EMI will be an interactive intervention allowing healthcare workers to record their emotional states at momentary periods. In addition, they will receive contextual ER strategies on request. Considering the recent research on ER strategy choice [49, 53, 66], the logic of displaying one ER strategy or another via EMI will be based on the type of emotional state and emotional intensity reported by the participant. On the other hand, because emotional awareness is a form of emotion regulation processing [66], assessing emotions daily may help improve the self-awareness of emotions, thus contributing to a better emotional regulation. Therefore, an EMA only condition will be included in this study to distinguish the effect of EMA and EMI. The EMI protocol will include different transdiagnostic cognitive behavioral interventions that will be administered to healthcare workers who are or have been recently and repeatedly exposed to COVID-19 related stress. The treatment will be administered via smartphone during 2 months. Treatment efficacy will be evaluated at post-treatment and 3 months later (follow-up). To the best of our knowledge, this is the first investigation that aims to evaluate the efficacy of an ecological, momentary, and self-administered transdiagnostic psychological intervention via smartphone for healthcare professionals during the pandemic.

## Methods/design

### Study aims and design

The goal of this study is to evaluate the efficacy of an ecological, momentary, and self-administered transdiagnostic psychological intervention via smartphone for healthcare professionals (compared to an ecological momentary assessment group and a wait-list group) to decrease depression, difficulties in emotion regulation and increase professional quality of life and resilience. We also would like to study the usability and acceptability of the technology.

**Fig. 1:**
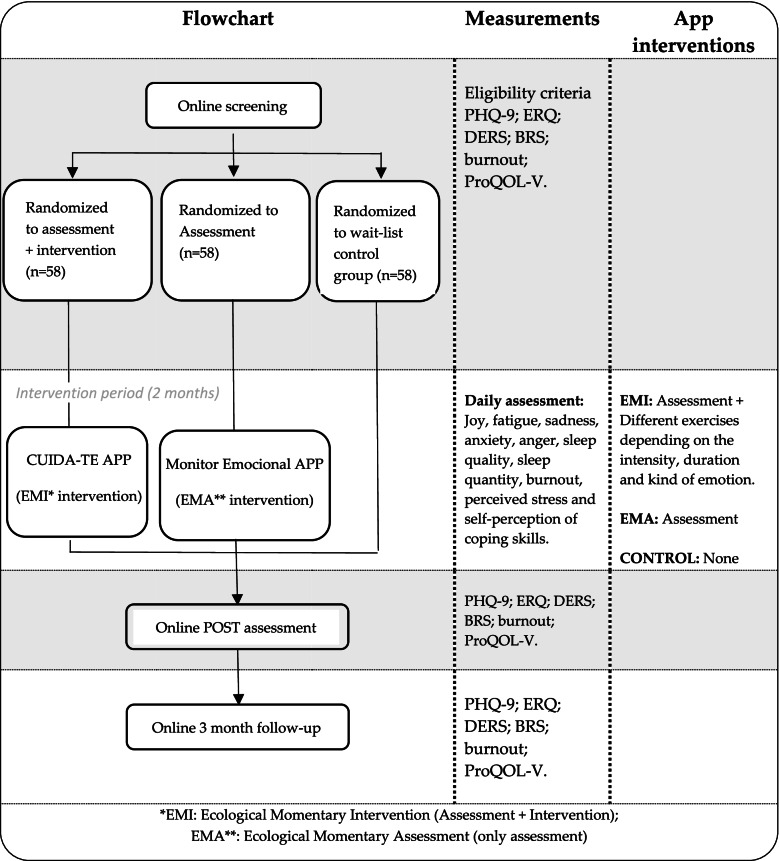
Design flowchart

The current study will use a randomized controlled superiority trial design. A minimum of 174 healthcare workers will be randomly assigned into three conditions: (1) EMI intervention group, (2) EMA intervention group, and (3) wait-list control group. An online informed consent form will be signed by the participants before randomization. Assessments will be conducted at pre-treatment, post-treatment, and 3- month follow-up. The experimental groups will use different apps during 2 months: the experimental group (1) will use an EMI app (CUIDA-TE) and the experimental group (2) will use an EMA app (MONITOR EMOCIONAL). For ethical reasons, when the wait-list group completes the post-treatment assessment (2 months after the base-line evaluation), the EMI app will be offered if the interim analyses support this. Participants in the EMA condition will also be offered this option at the end of the 3-month follow-up, again if the results support this. The trial has been approved by the Ethics Committee of Humans Research of the Ethics Commission in Experimental Research of University of Valencia (Spain) and it has been registered in clinicaltrials.gov as NCT04958941 (https://clinicaltrials.gov/ct2/show/NCT04958941). The study will adhere to the recommendations of the Consolidated Standards of Reporting Trials (CONSORT) [67]. The design of the study is outlined in Fig. 1.

### Participants, recruitment, and eligibility criteria

A sample of 174 healthcare workers will be recruited from the Spanish healthcare system (hospitals, primary care centers, and nurse care centers). Participants will be eligible if they meet the inclusion criteria: 1) being an active healthcare worker and 2) having an Android smartphone with Internet access. Participants will be excluded if they are already receiving a psychological treatment at the time of the recruitment or 2) having a smartphone with an operating system different to Android. Participants will be recruited from two sources: 1) volunteers from a large healthcare provider company (GESMED company, with 2.500 healthcare workers at the region) and 2) volunteers from different healthcare organizations recruited online using social media (paid campaigns) and personal contacts.

### Sample size

The sample size was calculated a priori using GPower [68]. Assuming a conservative effect size of 0.20, an alpha level of 0.05, an 80% power, three groups, and 3 measurements, a sample of 152 was obtained. Assuming a 15% of attrition, we will recruit a minimum of 174 participants. The small attrition rate is based on a systematic review showing that interventions for mental health that incorporate technology, such as blended interventions, tend to obtain lower dropout rates [69]. The effect size in favor of EMI compared to EMA and the control condition is also inspired by past research [70].

### Measures description

#### Primary and secondary outcomes

The sociodemographic information (see Supplementary file 1) will be gathered with an ad hoc questionnaire that evaluates sex, age, specific healthcare profession, marital status, if they share their home with a COVID-19 vulnerable person, where they work (type of health care center, such us hospital, primary care, nurse home, etc.), if they work directly with COVID-19 patients, and number of COVID-19 deaths in their region (this will be collected from external, official sources) [2].

The primary outcome is the Brief Patient Health Questionnaire Mood Scale (PHQ-9) for depression. Secondary outcomes include the Emotion Regulation Questionnaire (ERQ), the Difficulties in Emotion Regulation Questionnaire (DERS), the Brief Resilience Scale (BRS), and the Short Professional Quality of Life Scale (ProQOL Short). Primary and secondary outcomes will be obtained at baseline, post-intervention, and 3-month follow-up.

The PHQ-9 is a 9-item depression module from the full PHQ where the user indicates the severity of depression during the previous 2 weeks. The symptom is presented in a scale measuring the frequency from 0 to 3 (0= “not at all” to 3 =” nearly every day”). The PHQ-9 has demonstrated its validity as a brief measure of depression severity in clinical samples [71] and the general population [72]. The PHQ-9 score is divided into the following severity categories: 5–9 (mild depression), 10–14 (moderate depression), 15–19 (moderately severe depression), and 20 or greater (severe depression). The range for the PHQ-9 is 0-to-27. The Spanish version of the scale has demonstrated good psychometric properties [73].

The ERQ is a 10-item self-report measure that evaluates the use of cognitive reappraisal and emotion suppression as ER efforts to regulate emotions [74, 75]. The items are rated on a 7-point scale (1 = “strongly disagree” to 7 = “strongly agree”). We will use the Spanish adaptation of the ERQ [76], which has obtained good internal reliability estimates for both reappraisal (.79) and suppression (.75).

The DERS [77] is a 28-item questionnaire that explores five dimensions of emotion dysregulation, including lack of control, life interference, lack of emotional attention, emotional confusion, and emotional rejection. Items are rated on a scale of 1 (“almost never”) to 5 (“almost always”). We used its Spanish adaptation [78]. The Cronbach’s alpha of the total dysregulation score has been excellent: .93 [77].

The BRS [79] evaluates resilience, understood as the ability to recover from stress. The BRS has 6 items rated from 1 (“strongly disagree”) to 5 (“strongly agree”). The scale has a Cronbach’s alpha of .80.

The ProQOL Short [80] is a 9-item questionnaire with 3 subscales: Compassion satisfaction, which evaluates the positive and altruistic aspects of the helping work, burnout, and secondary traumatic stress. The responses are rated on a 5-point Likert scale, in which participants indicate the frequency of each item in the last 30 days (1 = “never”, 2 = “rarely”, 3 = “Sometimes”, 4 = “Often”, and 5 = “Very Often”). ProQoL Short is based on version V of the Professional Quality of Life Scale. This questionnaire has been recently used in many studies about psychological effects of COVID-19 in healthcare professional [9, 81, 82] and its subscales have obtained good internal consistency estimates ranging from .75 to .88 [83]. ProQoL Short showed an adequate internal structure and invariance across the studied countries [80].

In addition to the clinical measures, we will evaluate typical outcomes in technology studies, namely usability and acceptability of the technology and fidelity with its use. Usability and acceptability of the technology will be measured with the Acceptability and System Usability Scale (SUS) and the Usability and Acceptability Questionnaire (CUA-Brief). The SUS [84] is a reliable, 10-item questionnaire that assess the usability of a technology application. The SUS has an acceptable reliability (Cronbach alpha of.91) [85]. Items are rated from 1 (“Strongly disagree”) to 5 (“Strongly agree”). Unacceptable usability could indicate that a user had difficulties while using the program, thus suggesting that the administration of the intervention through technological application could be considered a barrier for the clinical effect. The SUS can be interpreted with a qualitative scale (from “worst imaginable” to “best imaginable”) [86]. The CUA-Brief measures the acceptability of a technology using 7 items. It was designed ad hoc in a previous study. This is not an officially validated measure, but its Cronbach alpha in a previous study by our team was excellent: .94 [87]. The CUA-Brief allows to assess the user’s opinion about an application (perceived control while using the application, perceived ease-to-use of the app, intention to use the application in the future, confidence when using the app, feelings when using the app, and satisfaction with the size of the elements). All items are rated on a 5-point Likert scale ranging from 0 (“Totally disagree”) to 4 (“Totally agree”).

Finally, fidelity will be evaluated using passive outcomes, in line with past research. These measures will include the percentage of users making daily logins (at least 1) with respect to the total number of participants who started the treatment, the Percentage of users who continue to use the APP after 4 weeks, the Percentage of users who voluntarily use the APP outside the scheduled hours, and the Total time spent using the APP [88].

#### Daily measures

As mentioned earlier, the daily assessment of emotions could improve self-awareness about the emotional status and thus contribute to better emotional regulation. To control for this, the two mobile applications (EMI app and EMA app) will assess the same variables daily. These will include joy, fatigue, sadness, anxiety, anger, burnout, sleep quantity, sleep quality, perceived stress, and implemented ER (coping) skills, which have been found to be relevant emotional states reported by healthcare workers [10, 11]. Each variable will be responded using an 11-point slider question (from 0 to 10). Table 1 contains a description of the items used in the study. Items from 1 to 5 have been previously validated in a previous study [58], while items 6 to 10 have been elaborated ad hoc for this investigation. All items use the same format, except item 7 (sleep quantity), where the user has to select the number of hours slept. The users will receive a daily reminder to answer the 10 questions. The purpose of this reminder is to facilitate adherence. However, following the real-time and real-world philosophy of ecological and momentary assessments, the users will be able to open the app and answer to these questions on demand at any moment.

**Table 1:** Daily assessment

Item	Construct assessed	Description
1^a^	Joy	Please, indicate the intensity of your CURRENT HAPPINESS 0 = No happiness-------------------------------10 = Extremely happy
2^b^	Fatigue	Please, indicate the intensity of your CURRENT FATIGUE 0 = No fatigue--------------------------------10 = Extreme fatigue
3^a^	Sadness	Please, indicate the intensity of your CURRENT SADNESS 0 = No sadness-------------------------------10 = Extreme sadness
4^a^	Anxiety	Please, indicate the intensity of your CURRENT ANXIETY 0 = No anxiety-----------------------------10 = Extremely anxious
5^a^	Anger,	Please, indicate the intensity of your CURRENT ANGER 0 = No anger-------------------------------10 = Extremely angry
6^b^	Burnout	Please, indicate your degree of agreement with the next statement: “I am burned out from my work” 0 = totally disagree----------------------------------------10 = totally agree
7^c^	Sleep quantity	How many hours have you slept in the last 24 h?
8^c^	Sleep quality	Has WORK interfered with the quality of your SLEEP tonight? 0 = No Interference----------------------10 = Maximum Interference
9^c^	Perceived stress	Please, indicate the intensity of your CURRENT STRESS 0 = No stress------------------------------10 = Extremely stressed
10^c^	Self-perception of coping skills	To what extent do you feel CAPABLE OF COPING with your problems right now? 0 = No capable---------------------------------10 = Extremely capable

Note: ^a^Items extracted from Suso-Ribera et al. [58]. ^b^Item inspired by Morgantini et al. [17]; ^c^Items elaborated ad hoc for the current study

### Interventions

Two active conditions will be used in this study, that is, (1) the EMI intervention and (2) the EMA condition. Because, Android OS represents 80% of the users in Spain [89], the apps for this study have been developed only in this operating system. Further development in other mobile operative systems will be done after the results have been analyzed.

#### Monitor emocional APP (EMA condition)

“MONITOR EMOCIONAL” is an app for ecological momentary assessment developed in Android OS (https://play.google.com/store/apps/details?id=monitoremocional.code&gl=ES). To use this app, users will have to obtain a code from the researcher. The app will be active during 2 months. When the users open the app, the 10 daily items from Table 1 will be showed in a linear manner. After the two-month daily evaluation, the app will suggest the users to uninstall the application and will stop sending them the reminders to complete the evaluations. The users will be able to use the app any moment during the two-month period. The intervention aspect of this app is grounded on the first step of the emotion regulation process, that is, self-awareness of emotions through daily assessment.

#### CUIDA-TE APP (EMI condition)

“CUIDA-TE” (which could be translated to “TAKE CARE OF YOURSELF”) is an app for EMI developed in Android. As in the previous app, to use the CUIDA-TE app the user will have to obtain a code from the researcher. The app will be active during 2 months. Again, when users open the app the 10 daily items from Table 1 will be showed in a linear manner. The interventions are mediated by the intensity and type of emotional state. Thus, when the user reaches the specific scores detailed in Table 2, an EMI will be activated to offer the intervention objects linked with this score and construct. The clinical alarms in Table 2 were set after a series of meetings with experts in clinical psychology and mood disorders. For the cut-offs of severe, moderate, and mild levels of symptomatology, we followed the recommendations from past research (0–4 for mild, 5–6 for moderate, and 7–10 for severe) [90]. Considering the results on ER strategy choice research [49, 53], when participants report a low-moderate emotional intensity, interventions related to cognitive reappraisal and behavioral change will be proposed. On the contrary, when participants indicate high emotional intensity, strategies focused on attentional deployment such as relaxation, mindfulness, and distraction will be indicated.

**Table 2:** Structure of the ecological momentary intervention

Item	Construct assessed	Activate EMI when item scores	Ecological momentary intervention (EMI)
1	Joy	*High:* from 7 to 10	Savoring
2	Fatigue	*High:* from 7 to 10	Psychoeducation in self-care Mindfulness 5 senses Relaxation-Paced breathing exercise
3	Sadness	*High:* from 7 to 10	Mindfulness in a joyful environment Reminiscence
		*Moderate:* from 5 to 6 during 2 consecutive days.	Psychoeducation of emotion’s function Checking the facts Behavioral activation (based on goals and values)
4	Anxiety	*High:* 7 to 10	Safe place exercise Relaxation-Paced breathing exercise Mindfulness in a relaxing environment
		*Moderate:* 5–6.	Cope ahead of time
5	Anger	*High:* 7–10	STOP exercise Mindfulness in a relaxing environment Relaxation-Paced breathing exercise Relaxation-Progressive Muscle relaxation exercise Safe place exercise
		*Moderate:* 5–6	Problem solving Acceptance with the body
6	Burnout	*High:* 7–10	Psychoeducation on self-care Mindfulness 5 senses Mindfulness in a relaxing environment
		*Moderate:* 5–6 during 3 consecutive days.	Behavioral activation (based on goals and values).
7	Sleep quantity	*Low:* 0–4 h during 2 consecutive days.	Psychoeducation: The need of self-care to be able to care others.
		*Moderate:* 5–6 h during 4 consecutive days	Psychoeducation on sleep habits Psychoeducation on self-care
8	Sleep quality	*High:* 7 to 10	Savoring
		*Moderate:* 5–6 during 2 consecutive days	Psychoeducation on sleep habits Mindfulness in a relaxing environment
9	Perceived stress	*HIgh:* 7 to 10	Safe place exercise Mindfulness in a relaxing environment Relaxation-Paced breathing exercise
		*Moderate:* 5–6 during 5 consecutive days.	Psychoeducation: The need of self-care to be able to care others. Behavioral activation (based on goals and values) Problem solving
10	Self-perception of coping skills	*Low:* from 0 to 3.	Cope ahead of time
		*Moderate: 5* or over during 2 consecutive days.	Problem solving Behavioral activation (based on goals and values)

The interventions detailed in Table 2 consist of treatment strategies and exercises based on principles of different evidence-based transdiagnostic cognitive behavioral interventions that aim to regulate the problematic emotion experienced. Most of the strategies were adapted and abbreviated from the UP [91] and DBT treatments to be delivered through a smartphone as an EMI (using multimedia objects). In the following lines we briefly describe the intervention developed for each emotion. As a note, psychoeducation components were included in several EMIs to introduce the background of the intervention.

##### 1.-joy

When joy is reported to be high, the goal would be to include interventions to up-regulate positive emotion (i.e., increase its intensity or duration), which has been associated with fewer symptoms of psychopathology and increased reports of life satisfaction and well-being [92]. Different exercises are proposed to practice focusing on positive such as *savoring* (i.e., focus our attention on what is positive in our day-to-day life).

##### 2.-fatigue

Fatigue usually refers to an impairment in task performance at an individual’s normal capacity. The major cause of fatigue for healthcare workers are disruptions of sleep and other health habits [93], which have been aggravated after the COVID-19 pandemic [9]. Therefore, when fatigue is high, participants will be first directed to read a psychoeducation self-care module to improve health habits. After that, other mindfulness (*observe with five senses*) and relaxation (*paced-breathing*) exercises from DBT will be delivered to increase awareness and a sense of calm, respectively [24].

##### 3.-sadness

Mindfulness and positive reminiscence interventions are proposed when sadness is high. Mindfulness practice has been associated with increases in happiness [94] and decreases in depression [95]. An example of a mindfulness exercise is to *observe a joyful environment* of a virtual nature scenery. Remembering positive personal past memories is another recommended exercise. However, when sadness is moderate, reappraisal, which leads to a decrease in negative emotion experience and expression [96], and behavioral activation are recommended. An example of a reappraisal exercise is *checking the facts*, a skill from DBT [24]. Exercises of *behavioral activation* to achieve personal goals and values are also recommended to decrease depression [24].

##### 4.-anxiety

Different exercises of distraction (*safe place exercise*), relaxation (*paced breathing exercise),* and mindfulness (*observing nature with relaxing instructions*) are proposed for high anxiety scores. The ultimate goal of these techniques in the context of this intervention is to induce relaxation and calm as ways to rapidly reduce emotional arousal [24]. For moderate anxiety scores, a cognitive change technique named *cope ahead* is proposed to develop effective strategies to manage anxiety ahead of time using imagination [24].

##### 5.-anger

When anger is high, distraction (*STOP exercise*), mindfulnes*s* (in a relaxing virtual nature), and relaxation (*paced breathing and progressive muscle relaxation*) exercises are proposed. An example of EMI for high anger is the *STOP skill* that instructs participants to step back, observe, and proceed mindfully towards the situation that sets up the emotion [24]. For moderate anger scores, *problem solving* is aimed to change difficult situations when an emotion fits with the facts [24], whereas acceptance refers to accepting the situation or incapacity to deal with it [97]. This last strategy is especially useful in situations that cannot be easily modified or reappraised (e.g., deaths that resulted from COVID-19) and has been found to be protective at both psychological and physical levels [97]. Examples of acceptance interventions are short exercises to practice *acceptance with the body* [24].

##### 6.-burnout

Research has shown that the COVID-19 pandemic has negatively impacted the feeling of burnout in healthcare professionals [9]. When burnout is high, as recommended with fatigue, participants will be first directed to a psychoeducational module. After that, mindfulness exercises to increase emotional awareness and a sense of calm will be proposed. On the contrary, when burnout is moderate, working on goals and values through behavioral activation exercises is recommended.

##### 7,8.-sleep quantity and quality

Disruption of the circadian sleep rhythm and sleep deprivation are common in healthcare professionals [93]. Both sleep quantity and quality will be measured. When sleep quantity is low or moderate (assessed as the number of sleep hours), psychoeducation on sleep and self-care habits are recommended. Regarding sleep quality, when this is high, participants will be instructed to practice *savoring* of the moment to increase awareness about the positive experience. On the contrary, when this is moderate, the participants will receive mindfulness and relaxation exercises to increase awareness and calmness.

##### 9.-perceived stress

This refers to the degree to which people evaluate life situations as stressful [98]. Research has shown that perceived stress has increased among health care professionals working during the COVID-19 [99]. In the study, when perception of stress is high, the same exercises proposed for anxiety will be recommended. As opposed to this, when stress levels are moderate, strategies to achieve behavioral change such as problem solving and behavioral activation will be proposed. In addition, a psychoeducational self-care module will also be presented for moderate stress.

##### 10.-Self-perception of coping skills

Research has revealed that positive coping strategies have a beneficial impact on mental health outcomes [100]. When self-perception of coping skills is low, practicing *cope ahead* can help healthcare professionals to develop and observe themselves practicing effective coping skills on the imagination in order to later put them into practice in real stressful situations. On the other hand, for moderate scores, problem solving and behavioral activation are recommended to develop solutions and take steps to achieve personal values respectively.

**Fig. 2:**
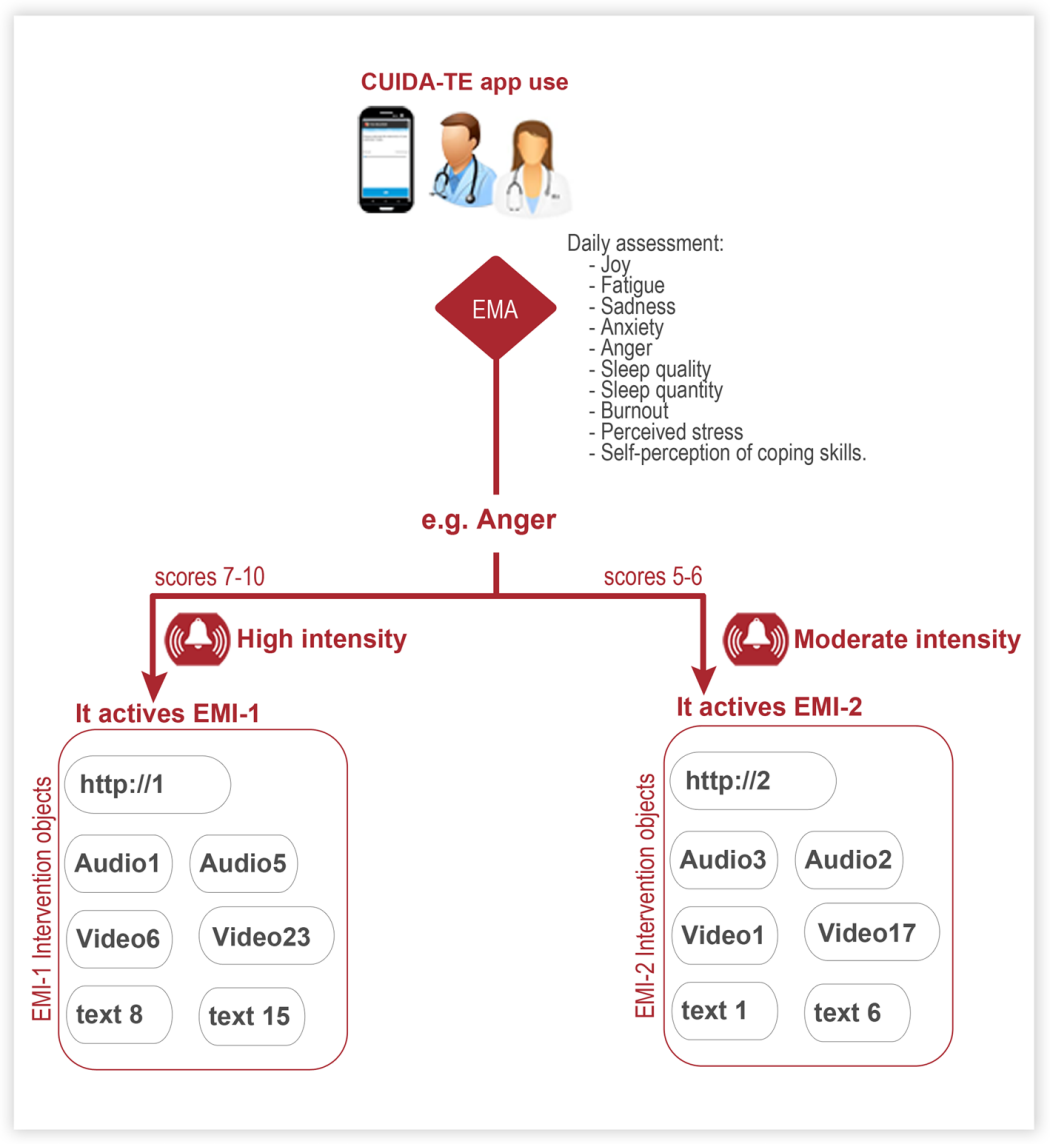
Example of EMI flow app for anger construct

Whenever a user scores within the parameters defined in Table 2, an EMI will be activated and the interventions will be presented in the form of exercises in multimedia and self-applied format (audio, video, text, or little webpages). The exercises will be stored in the library section, so that the user will be able to revise the content at will. An example of the flow followed by the users using the EMI app is shown in Fig. 2.

The intervention of the CUIDA-TE app is grounded on 1) self-awareness of emotions through daily assessment and 2) learning and training adaptive responses depending on the nature and intensity of the emotion.

**Fig. 3:**
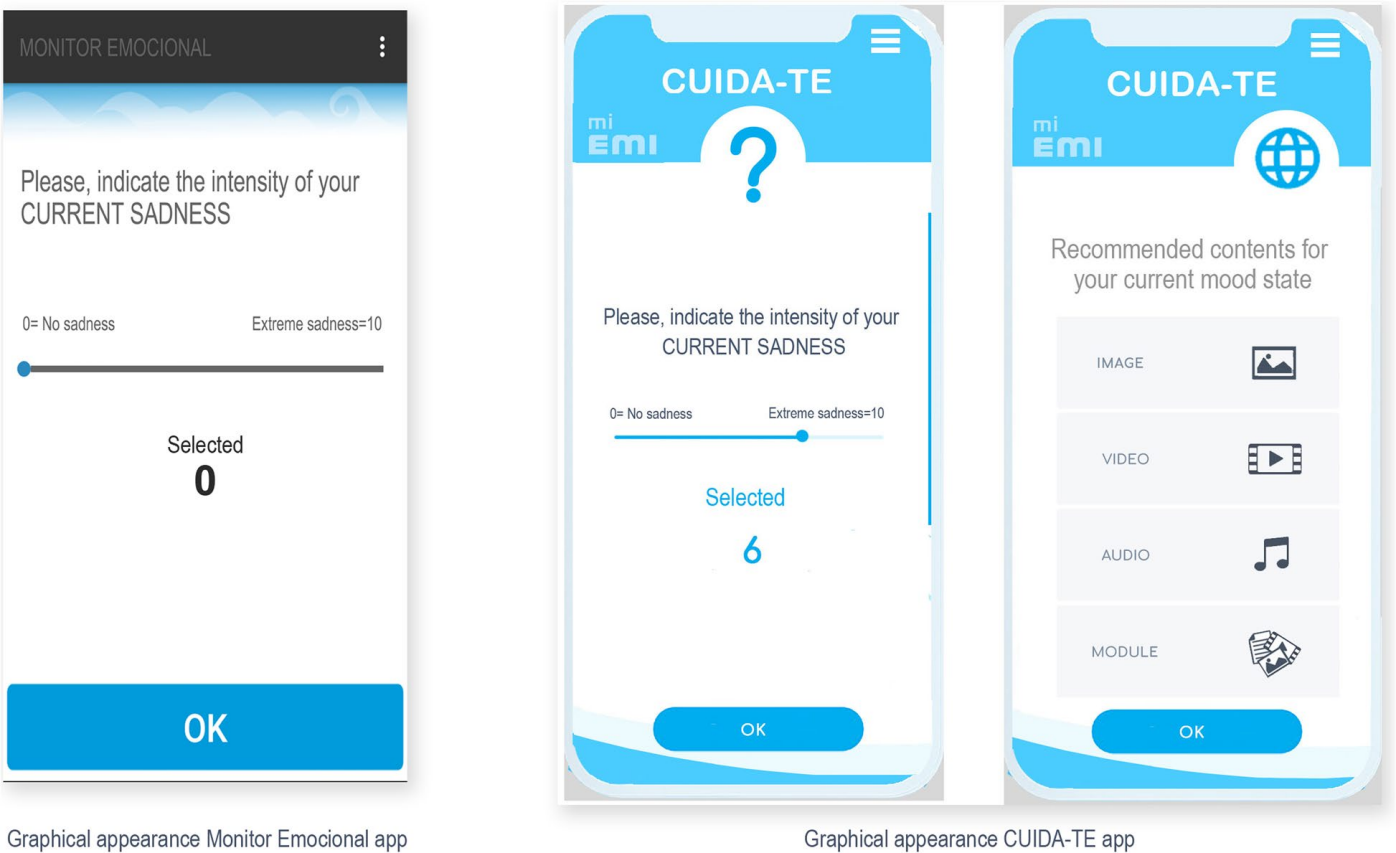
Graphical User Interface example of both applications

Shared features (the initial instructions and the assessment) from both apps also include an equivalent graphical user interface. Figure 3 shows an example of the graphical user interface of both apps. Both APPs have a missing alarm as well. If the user does not answer to the daily EMA during four consecutive days, the lead researcher will receive an automatic email warning and contact the user to check if they have a technical problem or if this reflects a potential dropout. This procedure aims to reduce attrition rates.

### Randomization and procedure

Participants will be volunteers of a healthcare provider company (GESMED) and different healthcare organizations (online recruitment). For volunteers recruited online, the call of participants will be made using an open campaign that will be disseminated via social networks (WhatsApp, Instagram, Twitter, Facebook, and LinkedIn) and personal contacts. For the GESMED volunteers, the call of participants will be done sending an internal e-mail to the workers offering the study information and the website for the study (www.cuidateapp.com). Interested candidates from both sources who meet the inclusion criteria will be included in the study after the informed consent is signed online. The randomization will be conducted by an independent researcher not involved in the study and qualified from clinical point of view. Participants will be allocated to one of the three conditions using a computer-generated random software (Random allocation software 2.0 [101];). After signing the consent form and once the randomization is made, the participants will know the condition in which they are allocated and will receive a brief explanation of the characteristics of their condition.

First, all users will fill the baseline questionnaires (primary and secondary outcomes) online. The first intervention group will use the CUIDA-TE app during 2 months. The second intervention group will use MONITOR EMOCIONAL app, again during 2-months. The wait-list control group will remain without treatment in a waiting list condition during 2-months. After that, they will be offered the opportunity to receive the intervention app (CUIDA-TE app) if the interim analyses support this. Primary and secondary outcomes will be measured again at post-treatment (2 months after the baseline assessment) and at follow-up (3 months).

## Ethics and dissemination

This study follows the ethical standards of the Declaration Helsinki and existing guidelines of Spain and the European Union for humans’ protection in clinical trials. All participants will be volunteers, and once the study has been explained to them and if they agree to participate, they will sign an online informed consent form. Participants will be able to withdraw from the study at any time. Regarding data security, both apps will follow the strictest rules, namely the General Data Protection Regulation (GDPR), Organic Law 3/2018 of 5 December and regulation 2016/679 of the European Parliament and of the Council [102] and neither CUIDA-TE nor MONITOR EMOCIONAL will store any personal data. The data will be stored on two servers connected locally at the University, following the corresponding legal regulations. However, the APP will not collect any personal data. The users will be identified with an external anonymous ID provided by the researcher. This information will only be known by the user and the lead researcher. This project, entitled “CUIDA-TE, an APP for the emotional management of the healthcare professionals” was approved by the Ethics Commission in Experimental Research of the University of Valencia in May 2021 (Register code: 1547727). It has been registered in clinicaltrials.gov (https://clinicaltrials.gov) as NCT04958941 (https://clinicaltrials.gov/ct2/show/NCT04958941).

## Data analysis plan

First, to ensure that the randomization process was successful, the three study conditions will be compared at baseline on all study outcomes. Then, a multilevel linear mixed model, which effectively handles missing data, will be computed. All assessment points will be included in the model. A restricted maximum likelihood method will be used to estimate the parameters. If differences at baseline are revealed, we will include them as covariates in the linear mixed model. Effect sizes (Cohen’s d) will be calculated [103]. Usability and acceptability will be evaluated with descriptive analyses.

All analyzes will be performed with SPSS v26.0. To conduct and report the study, we will follow the recommendations of the Consolidated Standards of Reporting Trials (CONSORT) [67].

## Discussion

Recent studies have indicated that the psychological effects of the pandemic COVID-19 among healthcare workers have been devastating globally [5]. In the same line, recent studies with Spanish healthcare professionals have shown a very high risk of developing a mental disorder (at least 50% of professionals) in this population [6, 11]. There is therefore an urgent call to action for mental health science to prioritize research on psychological interventions that can be feasibly delivered in the current pandemic conditions at a personalized level [38]. According to some studies, only one in four people receive psychological treatment and, in many cases, these interventions are not evidence-based [104, 105]. Public resources for mental health care are, in Spain and globally, clearly insufficient [106], which is reflected in long waiting lists to receive a first and successive appointments [107, 108]. Self-applied treatments via the Internet or with apps, which are at least as effective as active face-to-face individual therapy [41, 109–111], can reach the patient in a more economical and immediate way and are accessible to people who would otherwise have difficulties in receiving treatment (for example, due to long geographical distances to health centers, limited financial resources, stigma associated with mental health care, or lack of time). Therefore, these types of interventions are economically viable alternatives to face-to-face individual interventions, as they allow the dissemination of evidence-based treatments at low cost [43].

The use of ICTs in psychological treatments is not new. More than 100 randomized controlled trials have shown the effectiveness of online interventions [40], but the COVID-19 has undoubtedly boosted their progress, use, acceptability, and need due to the restrictions in delivering face-to-face interventions [112]. In this pandemic situation, the recommendations are to overcome the lack of time, the face-to-face restrictions, and facilitate personalized interventions in the moment when problems occur (or as close as possible).

Research has shown that ER allows individuals to cope with emotional situations [33]. Recent findings also revealed that ER strategies may not be adaptive or maladaptive per se, but depending on the context in which they occur [47, 48]. Thus, the use of traditional, contextually-unrelated interventions is likely to be insufficient. Ecological momentary interventions that include evidence-based ER strategies delivered in the context where the emotion occurs may be suitable to overcome all these barriers [113].

This paper describes the protocol of a randomized controlled trial that aims to investigate the efficacy of an EMI app based on principles of different transdiagnostic cognitive behavioral interventions focused on ER. Specifically, we want to investigate the efficacy of the CUIDA-TE app to improve the psychological well-being and reduce the suffering of healthcare workers who are or have been experiencing very stressful work situations related to the COVID-19 pandemic. It is known that stressful situations and chronic strains have a considerable impact on physical and mental health [114] and the literature supports the importance of training on adaptive strategies to regulate emotions in order to avoid negative psychological outcomes [113, 115, 116]. Therefore, the present study appears to fit well with the needs of healthcare professionals. If the APP proves to be useful for the present study sample, we intend to test its efficacy in different samples in need from the general population or in clinical samples with emotional disorders. Because daily emotional assessments only (i.e., EMA) could act as an emotional regulation intervention (because emotional awareness is linked with the emotion regulation process) [66], we will study the effect of daily emotional assessments (EMA condition with APP “*Monitor Emocional*”) and compare this with the effect of different transdiagnostic cognitive behavioral interventions (EMI condition with APP “*CUIDA-TE*”), as well as with a waiting-list condition.

The current study has a number of strengths. First, to our knowledge, this is the first RCT to test an ecological momentary intervention to enhance ER strategies on healthcare professionals. Second, using transdiagnostic interventions to prevent emotion dysregulation (instead of one intervention for each specific problem) allows us to manage different problems at the same time, thus reaching more people and saving costs and time for the healthcare systems. Third, this study will be carried in different health contexts (hospital, primary care, nurse homes, etc.) and professions (physicians, nurses, physiotherapists, etc.), which may provide a map of what types of professionals and contexts have endured the most extreme stress generated by the pandemic allowing us to design more specific interventions in the future.

Finally, some limitations are expected. First, taking previous literature, dropout rates are expected, so this has been considered in the sample size calculation. Second, stigma about mental health could exist among potential participants. To mitigate this, all the information in the app will be anonymous. It is also possible that some professionals are reluctant to participate. To address this, an information website currently under construction will be provided with detailed information about the study and previous experience of the team (http://www.cuidateapp.com/). Finally, because there is no active control (e.g., an onsite psychological intervention) because this is not offered in the Spanish health system for healthcare professionals in a routine manner, the comparisons will only be made between the waiting list condition and the two active comparators (EMI vs EMA).

## Conclusions

We expect that the findings of the study will contribute to advance in the knowledge on the use of EMAs and EMIs for emotion regulation. In addition, they will contribute to improve the quality of Internet-based psychological programs, which could be used for low-intensity interventions delivered through mobile phones or as complementary tools in face-to-face treatments.

## Trial status

Participant recruitment has not started. The recruitment will start in February 2022 and will continue approximately until October 2022. Follow-ups are expected to be completed by December 2022.

## Supplementary Information

**Additional file 1.**

## Data Availability

It is not possible to share the data because the study status is “Not yet recruiting”.

## Abbreviations

ACT: Acceptance and Commitment Therapy
APP: Application
BRS: Brief Resilience Scale
CONSORT: Consolidated Standards Of Reporting Trials
CUA-Brief: Usability and Acceptability Questionnaire
DBT: Dialectical Behavior Therapy
DERS: Difficulties in Emotion Regulation
EDs: Emotional disorders
EMA: Ecological Momentary Assessment
EMI: Ecological Momentary Intervention
ERQ: Emotion Regulation Questionnaire
ESM: Experience Sampling Method
ER: Emotion Regulation
GDPR: General Data Protection Regulation
ID: Identifier
ICTs: Information and Communication Technologies
OS: Operative System
PHQ-9: Brief Patient Health Questionnaire Mood Scale
ProQOL Short: Short Professional Quality of Life Scale
RCT: Randomized Controlled Trial
STOP: Stop; Take a step back; Observe; Proceed Mindfully
SUS: System Usability Scale
UP: Unified Protocol
