# Supplementary material for: Ecological momentary intervention to enhance emotion regulation in healthcare workers via smartphone: a randomized controlled trial protocol
Source: BMC Psychiatry. 2022 Mar 5;22:164. doi: 10.1186/s12888-022-03800-x (PMC8897724; doi:10.1186/s12888-022-03800-x)
Supplement: Supplementary file 1 — Additional file 1. [file 12888_2022_3800_MOESM1_ESM.docx]

Sociodemographic questionnaire

1. Age

2. Sex

3. Marital status

4. Work performed

a. *Medicine,*

*b. Nursing*

*c. Psychology*

*d. Physiotherapy,*

*e. Nursing assistant*

*f. Auxiliary*

*g. Laboratory technician*

*h. Pharmacy*

*i. Other _____________*

5. Type of facility

a. *Hospital,*

*b. Primary Care,*

*c. Socio-sanitary,*

*d. Nursing home,*

*e. Day care center,*

*f. Home care,*

*g. Ambulance,*

*h. Analysis laboratories.*

*i. Other______________*

6. Degree of contact with patients COVID-19

*a. None (I have not yet had contact with any positives),*

*b. Low (1-2 per month),*

*c. Moderate (1-2 per week),*

*d. High (7-10 per week),*

*e. Very high (daily).*

7. Do you share your home with a COVID-19 vulnerable person?

*a. Yes*

*b. No*

8. Degree of fear of self-infection

*0= No fear-------------------------------10= Extremely fear*

9. Degree of fear of infecting my family/friends

*0= No fear-------------------------------10= Extremely fear*
